# A giant isolated right coronary aneurism

**DOI:** 10.1259/bjrcr.20200208

**Published:** 2021-03-16

**Authors:** Chiara Andreoli, Emilia Biscottini, Johny Helou, Federico Crusco, Francesco Marchetti, Maurizio Scarpignato

**Affiliations:** 1 Department of Cardiology, S.G.Battista Hospital, Foligno (PG), Italy; 2 Radiology Department, S.M. della Misericordia Hospital, Perugia, Italy

## Abstract

A previously healthy 32-year-old female hailing from Mexico presented to the emergency department with rectorrhagia. Caseating granulomas were detected on histopathological analysis from cecum ulcerative lesions. A purified protein derivative skin test resulted positive. In order to exclude pulmonary tubercolosis, a CT lung scan was performed: a rounded and voluminous mass, located above the right atrioventricular cardiac junction, was unexpectedly revealed. Further, a cardiac magnetic resonance and a coronary angiography disclosed a giant (5 × 4,8 cm) isolated aneurysm of proximal right coronary artery with severe thrombotic layering. The patient was treated with isoniazid, rifampin, ethambutol, and pyrazinamide; after approximately 2 months of treatment, she had complete resolution of cecal lesions. Anticoagulant oral therapy with warfarin was started and the patient was submitted to coronary artery grafting bypass surgery.

## Clinical presentation

We describe a case of a previously healthy 32-year-old female hailing from Mexico presented to the emergency department with rectorrhagia. Her past medical history was silent. There was no family history of sudden cardiac death or cardiovascular disease. On physical examination, her vital signs were stable: heart rate was 80 beats/min, oxygen saturation was 98% in room air, and blood pressure was 120/70 mmHg. Chest examination showed equal chest expansion and equal vescicular breath sounds bilaterally. Cardiovascular examination showed no added sounds.

## Investigations/Imaging findings

**Figure 1.**
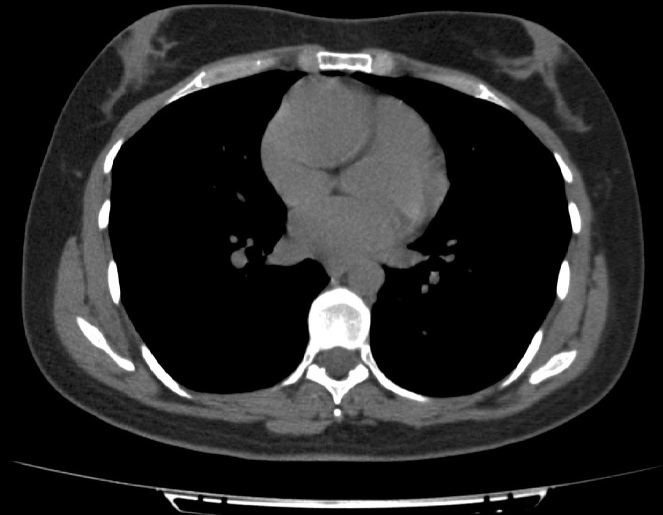
CT lung scan: isodence mass with no calcifications determining bulge on the right antero lateral cardiac border.

**Figure 2.**
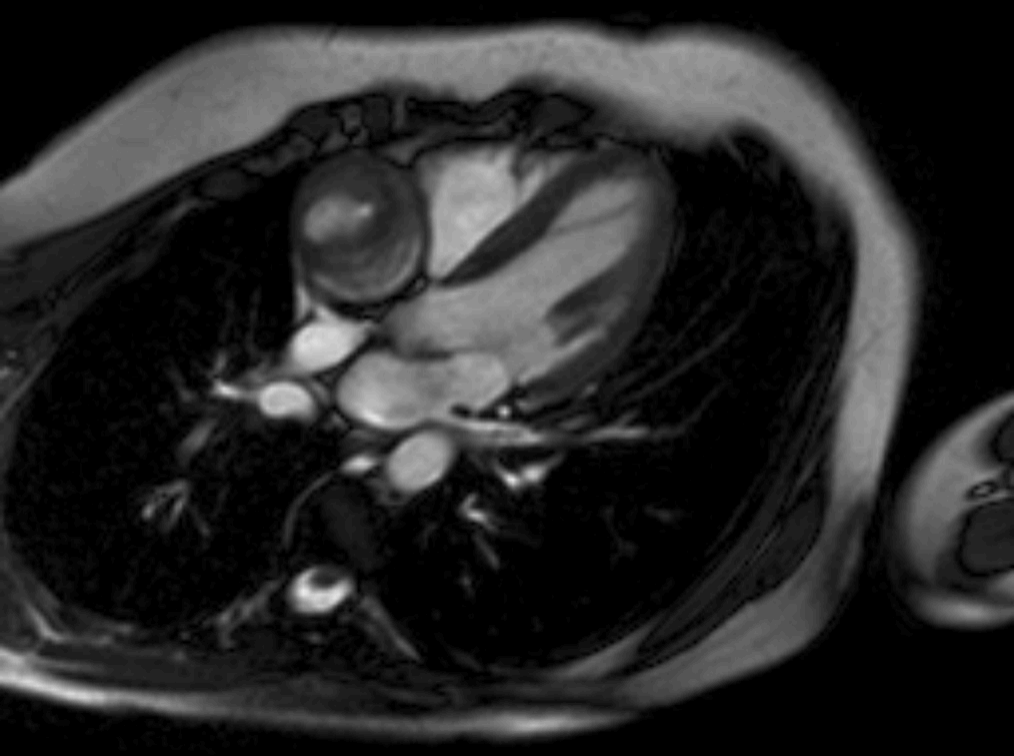
CMR four chamber: rounded ipointense mass with clear borders, central hyperintensity and concentric trombotic layering. CMR, cardiac magnetic resonance.

**Figure 3.**
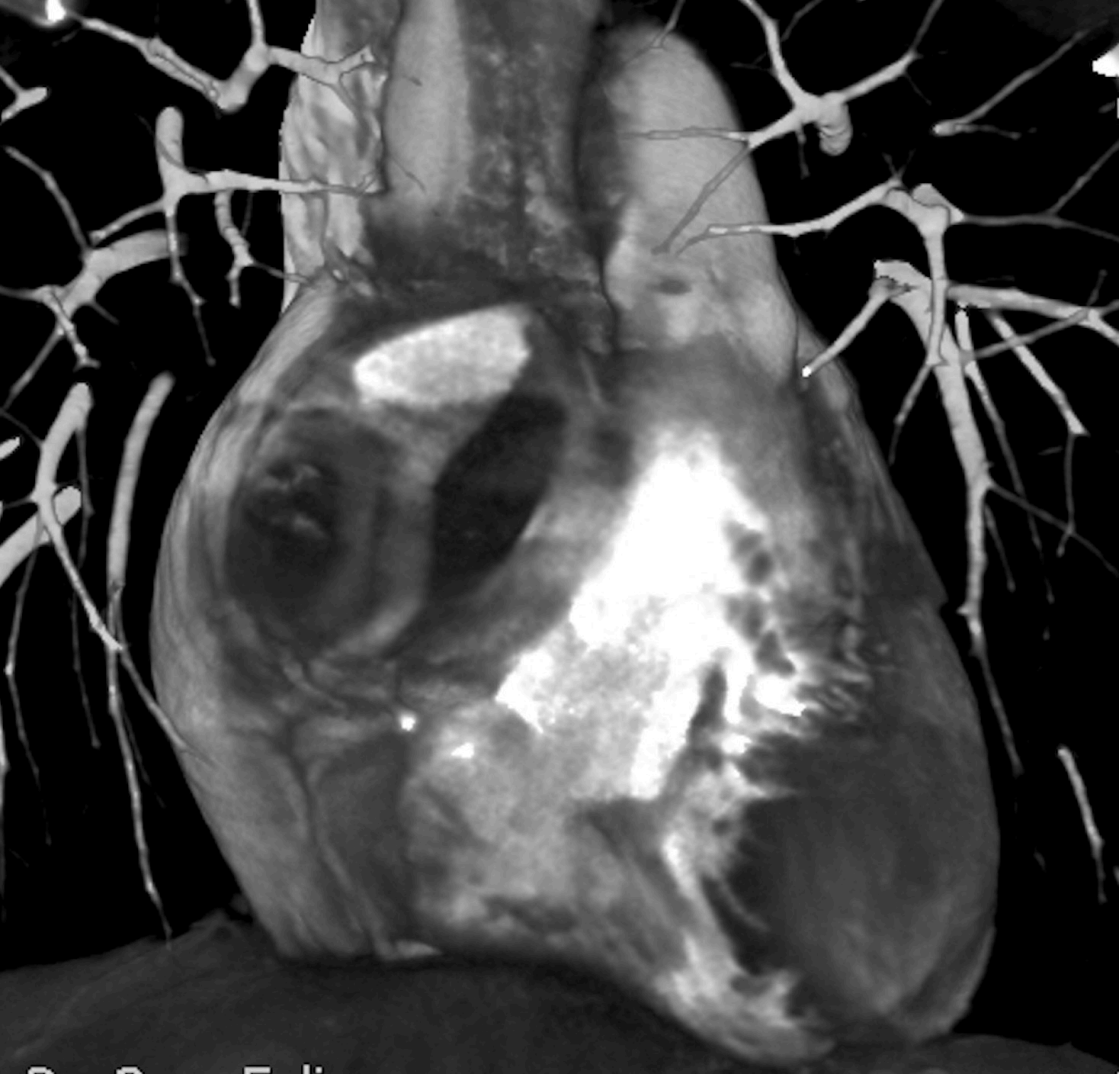
CT scan 2 MIP 3D: hypervascular mass of right coronary artery. 3D, three-dimensional; MIP, maximum intensity projection..

**Figure 4.**
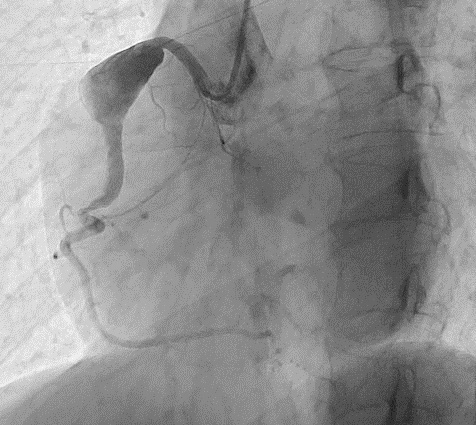
Coronary angiography: proximal right coronary artery aneurism with dilatation at the mid-tract, a 70% stenosis at the crux and TIMI three flow.

The blood exams were normal except for microcytosis. A chest X-ray was normal. A colonoscopy revealed cecum ulcerative lesions and an histopathological analysis was also performed. Further, an abdominal CT scan displayed parietal thickening of the cecal fundus associated with vegetative lesion protruding into the lumen and involving the ileocecal valve, mucosal thickening inducing secondary stenosis of the lumen and mesenteric adenopathies in right iliac fossa. Caseating granulomas were detected on histopathological analysis from cecum ulcerative lesions. Hence, the patient underwent a purified protein derivative skin test that resulted positive. In order to exclude pulmonary tubercolosis, a CT lung scan was performed: a rounded and voluminous mass, located above the right atrioventricular cardiac junction, was unexpectedly revealed (Figure 1, Supplementary Video 1 ). We immediately performed an echocardiogram as an initial investigation, but wasn’t useful in the differential diagnosis. Further investigations including first a cardiac magnetic resonance (Figure 2, Supplementary Video 2), a CT scan with contrast (Figure 3) and consequently a coronary angiography (Figure 4) disclosed a giant (5 × 4,8 cm) isolated aneurysm of proximal right coronary artery with severe thrombotic layering, dilatation at the mid tract, a 70% stenosis at the crux and TIMI three flow.

Supplementary Video 1.

Supplementary Video 2.

## Treatment

Based on the histopathological analysis results and the positive purified protein skin test, a presumptive diagnosis of tubercular coronary aneurysm was made; therefore the patient was treated with isoniazid, rifampin, ethambutol, and pyrazinamide for 2 months, followed by isoniazid and rifampin for 4 months. After approximately 2 months of treatment, she had complete resolution of cecal lesions but the cardiac mass remained the same. Therefore, considering the thrombotic layering and after the resolution of rectorrhagia, anticoagulant oral therapy with warfarin was started and the patient was submitted to surgical exclusion ofcoronary artery aneurysm (CAA) and right internal mammary artery bypass graft.

## Follow-up

At a follow up of 36 months, the patient is going well.

## Discussion

This case shows an occasional finding of giant CAA during a bleeding episode due to intestinal tuberculosis. Coronary artery abnormalities may be acquired or congenital. Giant CAAs are rare, with a reported prevalence of 0.02–0.2%.^1^ Causative factors may be atherosclerosis, Takayasu arteritis (TA), congenital disorders, Kawasaki disease, and percutaneous coronary intervention^2^; in particular a connection between tuberculosis and TA has been suggested.^3^ Tuberculosis arteritis usually results from direct spread of infection from adjacent tuberculosis tissues; obliterative granulomatous endarteritis is known in tuberculous meningitis, pulmonary tuberculosis and in the coronary arteries in tuberculosis pericarditis and myocarditis.^4^ Unfortunately, in our case, histopathological analysis from coronary aneurism was not performed, but given that a diagnosis of intestinal tuberculosis was made and considering that our patient neither had childhood history suggestive of Kawasaki disease, nor there was evidence of acute inflammatory disease or other acquired causes of CAA, a presumptive diagnosis of tubercular coronary aneurysm was made. Clinical sequelae of giant CAAs include thrombus formation, distal embolization of those thrombi, fistula formation, and rupture.^2,5^ A frequent finding is the presence of thrombus within the aneurysm.^2^ Non-invasive tools, including echocardiography, CT, and MRI, can detect some CAAs; anyway coronary angiography remains the gold-standard providing important information about the size, shape, location, and number of aneurysms.^6^ Most giant CAAs are asymptomatic, but some patients may present with angina pectoris, sudden death, fistula formation, pericardial tamponade, compression of surrounding structures, or congestive heart failure.^5^ Surgical correction is generally accepted as the preferred treatment for giant CAAs,^1,6^ however, the management in the absence of acute coronary syndrome is still not known. In the absence of surgical correction, some authors support the use of antiplatelet or antithrombotic treatment, or both, for all large aneurysms, in order to reduce the risk of *in situ* thrombus or distal embolization.^5^ It is now possible to treat selected patients with percutaneous techniques that are less invasive; however, long-term outcomes are still unknown.^7^

## Learning points

- Cardiac masses are not infrequent, rarely due to a CAA.
- Some cases describe association between tuberculosis and coronary artery inflammation resulting CAAs.
- Non-invasive tools can detect some CAAs, but coronary angiography remains the gold-standard.
- Surgical correction is generally accepted as the preferred treatment for giant CAAs, however, the management in the absence of acute coronary syndrome is still not known.
