# Supplementary figures and images for: A giant isolated right coronary aneurism
Source: BJR Case Rep. 2021 Mar 16;7(4):20200208. doi: 10.1259/bjrcr.20200208 (PMC8749390; doi:10.1259/bjrcr.20200208)

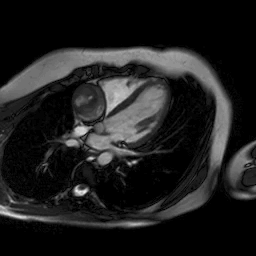

Supplement: Supplementary Video 2. [file bjrcr.20200208.suppl-02.gif]
